# Diagnostic value of immune-related biomarker FAM83A in differentiating malignant from benign pleural effusion in lung adenocarcinoma

**DOI:** 10.1007/s12672-024-01109-7

**Published:** 2024-06-24

**Authors:** Hangfeng Liu, Jia Yao, Yulan Liu, Liping Wu, Zhiwei Tan, Jie Hu, Shigao Chen, Xiaolin Zhang, Shuanghua Cheng

**Affiliations:** 1 The Second Affiliated Hospital of Chengdu Medical College, China National Nuclear Corporation 416 Hospital, Chengdu, 610051 China; 2 West China Biomedical Big Data Center, West China Hospital, Sichuan University, Chengdu, 610051 China; 3 Med-X Center for Informatics, Sichuan University, Chengdu, 610051 China

**Keywords:** Malignant pleural effusion, Lung adenocarcinoma, FAM83A, Immune infiltration, Biomarker

## Abstract

**Background:**

Malignant pleural effusion (MPE) is frequently observed in patients with advanced lung adenocarcinoma (LUAD). Pleural fluid cytology is a less invasive procedure compared to pleural biopsy. Therefore, it is crucial to identify novel effective biomarkers for LUAD-associated pleural fluid cytology.

**Methods:**

The RNA sequencing (RNA-Seq) and clinical data of LUAD cases were downloaded from TCGA and OncoSG databases. Differential gene expression analysis, survival analysis and immune cell infiltration analysis were performed on the LUAD datasets. The expression levels of FAM83A, TFF-1, and NapsinA in 94 paired LUAD and adjacent normal tissues, and in the pleural effusion specimens of 40 LUAD and 21 non-neoplastic patients were evaluated by immunohistochemistry.

**Results:**

FAM83A expression levels were significantly different between the LUAD and normal tissue datasets, and correlated with overall or disease-free survival, and histological grade of the tumors. Furthermore, the in-situ expression of FAM83A was higher in 89/94 LUAD tissues compared to the paired normal tissues. FAM83A expression was significantly correlated with immune cell infiltration, and showed a positive association with macrophage infiltration. In addition, FAM83A staining was positive in 37 LUAD pleural effusion samples, and negative in 20 non-neoplastic pleural effusion samples. The expression pattern of FAM83A in the pleural effusion of LUAD patients was relatively consistent with that of TFF-1 and NapsinA, and even stronger in some specimens that were weakly positive or negative for TTF1/NapsinA.

**Conclusions:**

FAM83A is a promising immune-related biomarker in LUAD biopsy specimens and pleural fluid, and can distinguish between malignant and benign pleural effusion.

## Introduction

Lung cancer remains the leading cause of cancer-related mortality worldwide. Non-small-cell lung cancer (NSCLC) and small-cell lung cancer (SCLC) respectively account for 85% and 15% of all lung cancer cases [1], and lung adenocarcinoma (LUAD) is the major histological subtype of NSCLC [1]. Malignant pleural effusion (MPE) is a common clinical complication of lung cancer, with a prevalence of 10-15% in advanced NSCLC patients at initial diagnosis, and a higher rate later during treatment [2]. Once the diagnosis of MPE is confirmed, NSCLC patients are classified into advanced TNM stages with a median overall survival of 5.5 months [3]. Therefore, accurate distinction between MPE and benign pleural effusion is critical for early clinical intervention and adequate management.

MPE is primarily diagnosed by pleural fluid cytology and pleural biopsy. Pleural biopsy is an invasive procedure that may result in various complications including hemorrhage and infection. In contrast, pleural fluid cytology is a less-invasive method, albeit with lower sensitivity and specificity compared to pleural biopsy. In fact, MPE diagnosis is undefined in some NSCLC patients due to negative cytology. Several biomarkers have been identified in pleural fluid cytology in recent years, including lactate dehydrogenase (LDH), carcinoembryonic antigen (CEA), carbohydrate antigen 153 (CA153) and adenosine deaminase (ADA) [4–6]. In addition, novel biomarkers such as reactive oxygen species modulator 1 (ROMO1), hyaluronic acid (HA), chitinase-3-like protein 1 (YKL-40), thymidine kinase 1 (TK1), pro-cathepsin D, and programmed death-ligand 1 (PD-L1) can improve the diagnostic ability of pleural fluid cytology, [7–12]. TFF-1 is highly expressed in lung adenocarcinoma, while Napsin A exhibits high specificity and sensitivity in lung adenocarcinoma [13]. It is often used in combination with TTF-1 to enhance diagnostic accuracy. Studies have shown that the combined detection of these two markers can effectively differentiate lung adenocarcinoma from other types of lung cancer and help distinguish between malignant and benign pleural effusions [14, 15]. To our knowledge, there is no consensus regarding the most effective biomarker for distinguishing MPE from benign pleural effusion, thereby warranting the exploration of new markers.

In this study, we screened the differentially expressed genes (DEGs) between the LUAD and para-cancerous tissues from the transcriptomic data in TCGA database, and identified family with sequence similarity 83 member A (FAM83A) as a potential diagnostic biomarker for differentiating malignant from benign pleural effusion in LUAD patients. Moreover, FAM83A may have an immunomodulatory function during the development of LUAD.

## Materials and methods

### FAM83A expression profile and clinical features in patients with LUAD

The RNA sequencing (RNA-Seq) and clinical data of LUAD were downloaded from cBioPortal for Cancer Genomics (http://www.cbioportal.org). The LUAD-2 (TCGA, Firehose Legacy), LUAD-3 (TCGA, Nature 2014) and LUAD-4 (TCGA, PanCancer Atlas) datasets from TCGA, consisting of 517 LUAD and 59 adjacent normal tissues, were integrated. The LUAD-1 (OncoSG, Nat Genet 2020) dataset included 169 Chinese LUAD samples with clinical information. The DEGs between 517 LUAD and 59 normal samples were screened using the DESeq2 R package (https://bioconductor.org/packages/release/bioc/html/DESeq2.html), with *p* value < 0.01 and |log2 fold change| ≥ 1 as the cut-off criteria. The patients in TCGA-LUAD were stratified into the FAM83A^high^ and FAM83A^low^ groups based on median expression level, and the differences in overall survival (OS) and disease-free survival (DFS) between the two groups were evaluated by the log-rank test. The hazard ratios with 95% confidence intervals and log-rank *P*-values were calculated. FAM83A expression levels in the well-, moderately-, and poorly differentiated tumors in the OncoSG dataset were compared using Student’s t test.

### Correlation of FAM83A expression and immune cell infiltration

Immune cell infiltration in each sample of TCGA-LUAD dataset was evaluated using the immunedeconv R package (https://grst.github.io/immunedeconv), which integrates the TIMER, CIBERSORT, EPIC, MCP-counter, quanTIseq and xCell algorithms. The correlation among different types of immune cells, and that between FAM83A expression and the infiltration of each immune cell type were determined by calculating Spearman’s correlation coefficient using the ggClusterNet R package (https://github.com/taowenmicro/ggClusterNet/). *P* value < 0.05 was considered statistically significant.

### Patient information and clinical samples

The experiments utilizing human tissue specimens complied with all the relevant national regulations and institutional policies, and were in accordance the tenets of the Helsinki Declaration. The study protocol was approved by the Ethics Committee of the Second Affiliated Hospital of Chengdu Medical College, China National Nuclear Corporation 416 Hospital. All clinical specimens were collected at the hospital, and informed consent was obtained from all subjects. Primary tumor specimens and paired normal lung tissues were obtained from 94 LUAD patients during surgical resection. The inclusion criteria were as follows: (a) definite diagnosis of LUAD, (b) absence of other malignant tumors, (c) TNM stages I-IV as per American Joint Committee on Cancer (AJCC) classification, and (d) availability of complete clinical and pathological data including age, sex, TNM stage, and clinical stage. In addition, pleural effusion was collected from 40 LUAD patients to 21 non-neoplastic patients. The inclusion criteria for LUAD patients were (a) pathologically confirmed LUAD and (b) absence of other malignant tumors. The inclusion criteria for the non-neoplastic subjects were (a) absence of malignant tumors and (b) lack of cancer cells in the pleural effusion.

### Immunohistochemistry (IHC)

Tissue specimens were fixed in 4% phosphate-buffered paraformaldehyde and embedded in paraffin. Fresh pleural effusion was left undisturbed for 20–30 min, and the sediment was collected and centrifuged at 2500 rpm for 5 min. After discarding the supernatant, the precipitate was successively washed with 75% and 95% ethanol, and then fixed in 10% formalin for 2–4 h. The solid cell pellet was dehydrated and processed with the same steps used for histological specimens. The paraffin blocks were cut into 3 µm-thick sections and subjected to routine immunohistochemistry. The specimens were probed with anti-FAM83A (Proteintech, Chicago, #20618-1-AP), anti-TTF1 (MXB^®^Biotechnologies, Fuzhou, China, #MAB-0677) and anti-Napsin A (MXB^®^Biotechnologies, Fuzhou, China, #MAB-0704) antibodies. Rabbit pre-immune serum was used as the negative control for primary antibody. Color was developed using diaminobenzidine (DAB) substrate and the sections were counterstained with hematoxylin. The sections were observed using the Olympus BX53 microscope. Staining intensity was scored as follows: 0 (negative) = negative staining; 1 (weak positive) = pale yellow; 2 (positive) = brownish-yellow; and 3 (strong positive) = brown. Patients with scores of 0 and 1 were classified in the low expression group, and those with scores of 2 and 3 were classified in the high expression group.

### Hematoxylin and eosin staining (H&E staining)

Tissue specimens were fixed in 10% neutral-buffered formalin and embedded in paraffin. Three micrometer paraffin sections were deparaffinized and heat-treated with citrate buffer (pH 6.0) for 7 min following an epitope retrieval protocol. Tissue sections were stained with hematoxylin and eosin (H&E) to facilitate the histological examination of tissue morphology and to identify any pathological changes.

### Statistical analysis

SPSS software (version 26.0; IBM, Chicago, IL) was used for statistical analysis. Chi-square test was used to analyze the correlation between clinicopathological characteristics and FAM83A expression. *P* value < 0.05 was statistically significant.

## Results

### FAM83A is a potential diagnostic and prognostic biomarker in LUAD

**Fig. 1:**
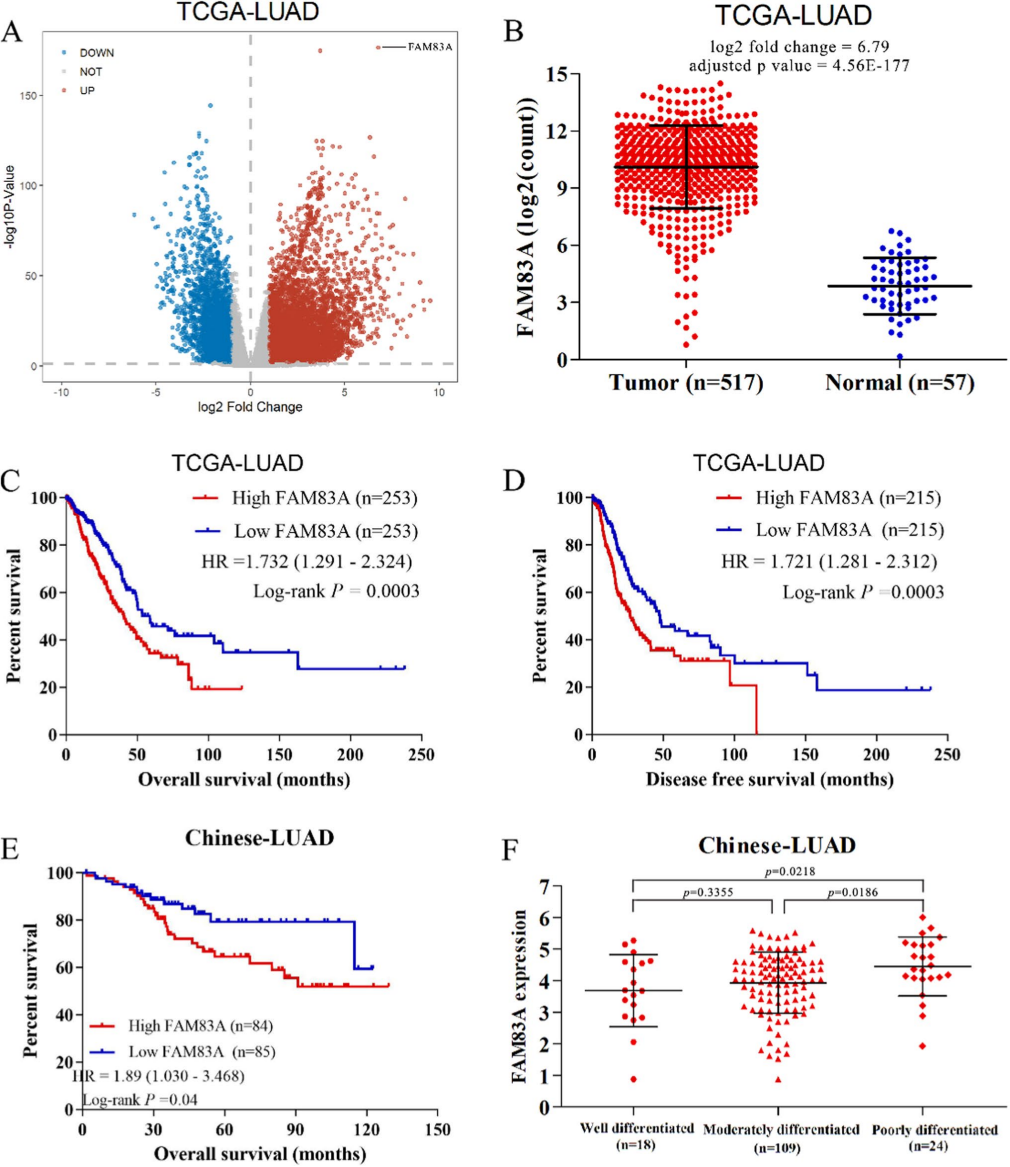
FAM83A is overexpressed in LUAD and associated with poor prognosis. **A** The volcano plot of FAM83A mRNA expression in the tumor and para-tumor samples in TCGA-LUAD dataset. **B** Relative expression of FAM83A in tumor and normal tissues in TCGA-LUAD datasets. **C** OS of FAM83A^high^ and FAM83A^low^ groups in TCGA-LUAD dataset. **D** DFS of FAM83A^high^ and FAM83A^low^ groups in TCGA-LUAD dataset.** E** OS of Chinese LUAD patients stratified by FAM83A expression in the OncoSG-LUAD dataset. **F** Difference in FAM83A expression between well-, moderately-, and poorly differentiated groups in the OncoSG-LUAD dataset

To identify biomarkers for differentiating between malignant and benign pleural effusion in LUAD patients, we first screened for the DEGs between 517 tumors and 59 normal tissues in TCGA datasets. FAM83A expression showed significant difference between the two groups, with log_2_ fold change 6.79 and adjusted *p* value 4.56E^−^177 (Fig. 1A). As shown in Fig. 1B, FAM83A was significantly up-regulated in LUAD tissues compared to the normal tissues, indicating its favorable discrimination ability. Furthermore, LUAD patients with high FAM83A expression had worse OS and DFS relative to those with low FAM83A expression (Fig. 1C, D). We also analyzed the relationship between FAM83A expression and the OS or histological grade of 169 Chinese LUAD patients. As expected, the FAM83A^high^ group exhibited shorter OS compared to the FAM83A^low^ group (Fig. 1E). Moreover, FAM83A expression level was higher in the poorly differentiated LUAD tumors that that in well- or moderately differentiated LUAD (Fig. 1F). Collectively, these findings suggest that FAM83A is upregulated in LUAD tissues and correlates with poor prognosis, indicating its ability to distinguish between tumor and normal lung tissues.

### High FAM83A expression correlated with poor pathological features

**Fig. 2:**
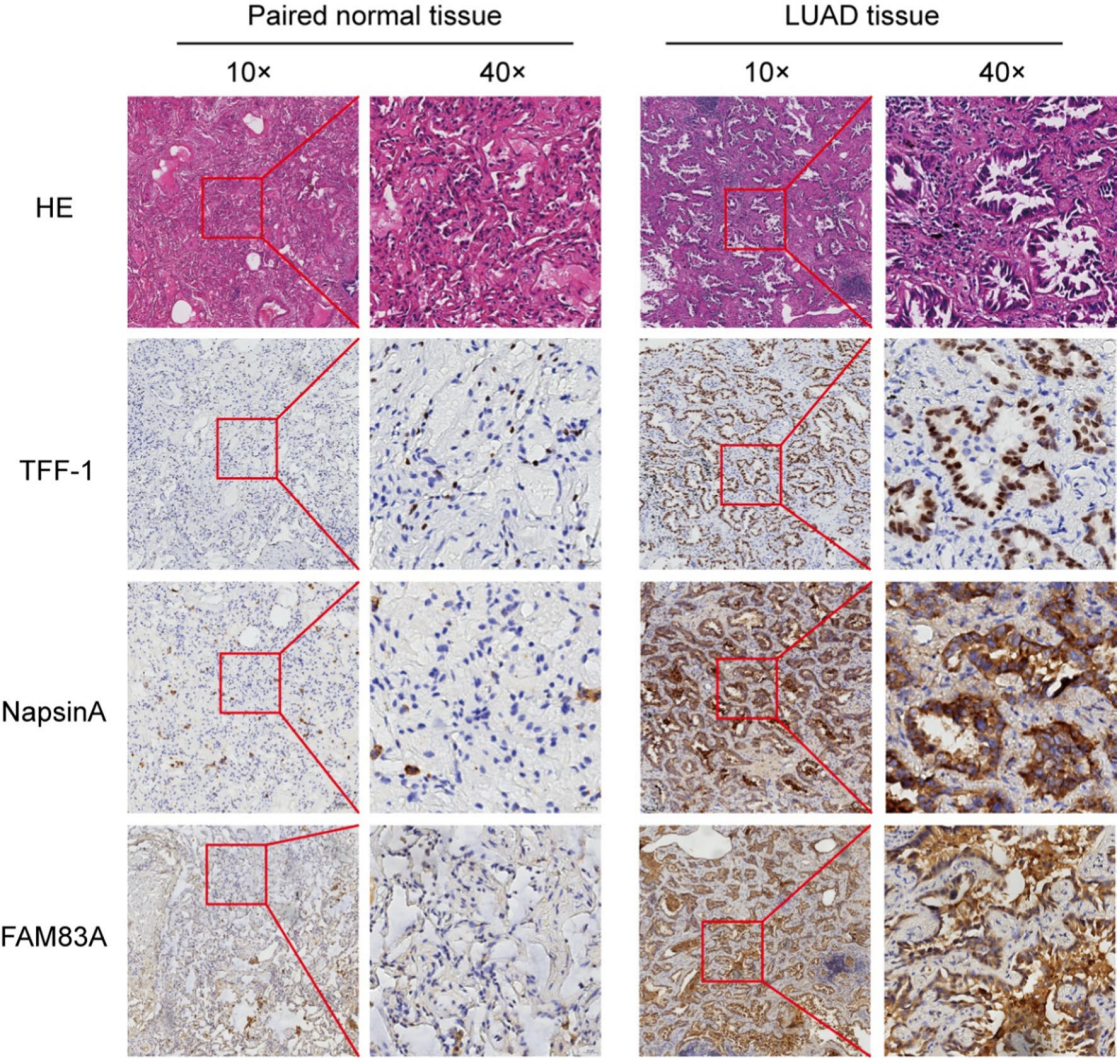
Representative images of FAM83A, TFF-1 and NapsinA immunostaining in LUAD and paired normal lung tissues

We quantified FAM83A protein expression in 94 LUAD and paired normal lung tissues by IHC. The FAM83A protein was detected in 89 (94.7%) LUAD specimens (Table 1, Fig. 2), and its expression pattern was relatively consistent with that of the common LUAD markers TFF-1 and NapsinA (Fig. 2). According to IHC score, we divided the 94 LUAD patients into the FAM83A^high^ (n = 64) and FAM83A^low^ (n = 30) groups. High FAM83A expression correlated significantly with T classification (*p* < 0.001), lesion size (*p* < 0.001) and pleural invasion (*p* < 0.001) (Table 2). The other clinical parameters, including age, gender, AJCC clinical stage, M classification and N classification, showed no significant difference between the two groups (Table 2). Taken together, high FAM83A expression correlates with poor pathological features and is a potential diagnostic marker for LUAD.

**Table 1:** FAM83A protein expression in 94 LUAD tissues and paired normal tissues

Group	n	Positive	Negative	Positivity rate	χ^2^	*P*
Paired normal tissue	94	3	91	3.2%	157.433	< 0.001
LUAD tissue	94	89	5	94.7%

**Table 2:** Correlation between FAM83A and clinical characteristics of LUAD

Clinical characteristics	n	FAM83A expression (%)		χ^2^	*P*
		Low expression	High expression		
Gender					
Male	40	9 (22.5)	31 (77.5)	2.840	0.092
Female	54	21 (38.9)	33 (61.1)		
Age (years)					
≤ 50	17	7 (41.2)	10 (58.8)	0.819	0.365
> 50	77	23 (29.9)	54 (70.1)		
Lesion size					
≤ 10 mm	15	12(80.0)	3(20.0)	18.992	< 0.001
> 10 mm	79	18(22.8)	61(77.2)		
Pleural invasion					
No	62	29 (46.8)	33 (53.2)	18.506	< 0.001
Yes	32	1 (3.1)	31 (96.9)		
Stage					
I + II	93	30 (32.3)	63 (67.7)	0.474	0.491
III + IV	1	0 (0.0)	1 (100.0)		
T classification					
T1 + T2	73	30 (41.1)	43 (58.9)	12.676	< 0.001
T3 + T4	21	0 (0.0)	21 (100.0)		
M classification					
M0	93	30 (32.3)	63 (67.7)	0.474	0.491
M1	1	0 (0.0)	1 (100.0)		
N classification					
N0	89	30 (33.7)	59 (66.3)	2.475	0.116
N1	5	0 (0.0)	5 (100.0)		

### FAM83A expression correlated with immune cell infiltration in LUAD

**Fig. 3:**
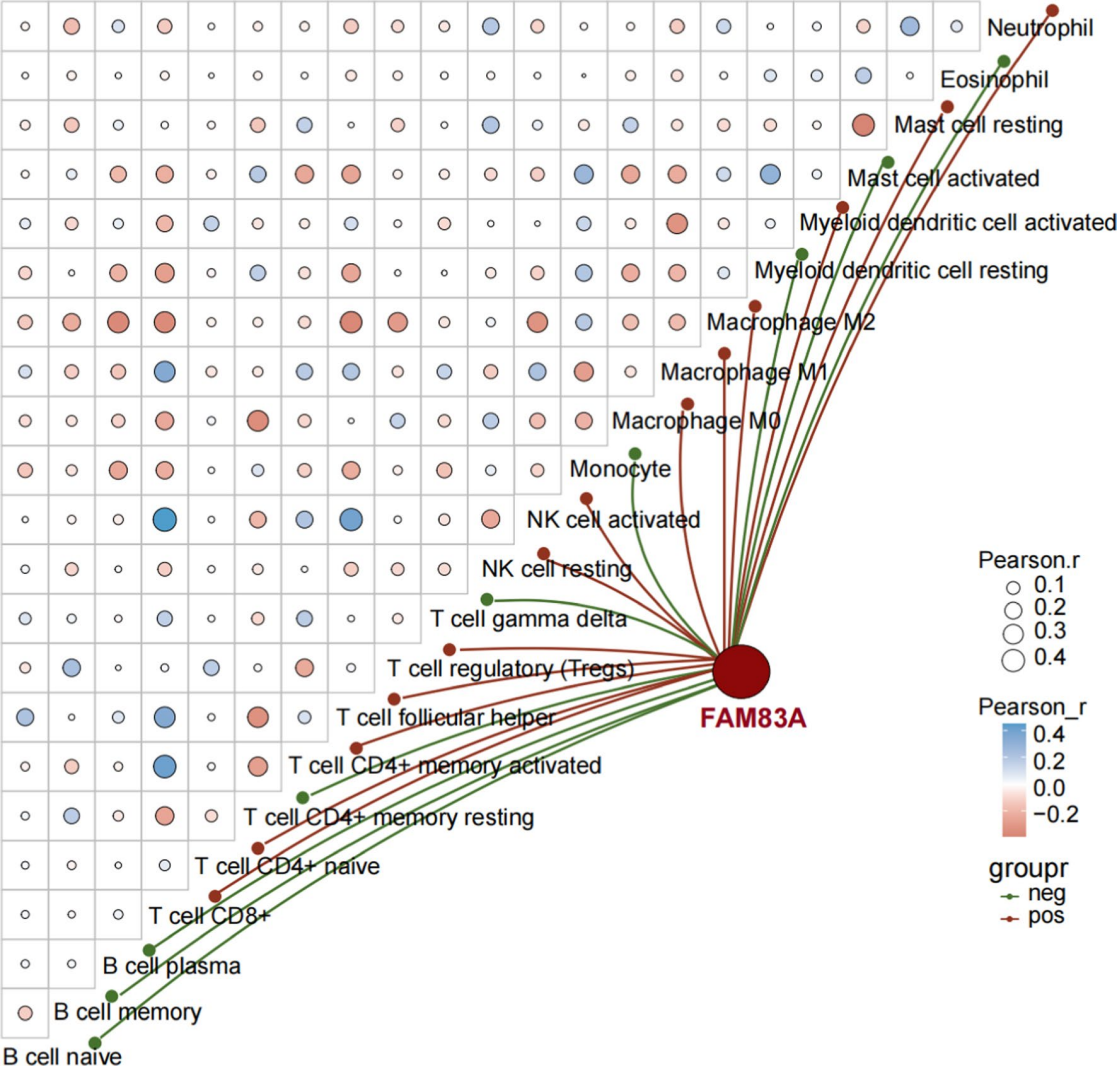
Correlation between FAM83A expression and immune cell infiltration in TCGA-LUAD. The heatmap shows the association among 22 immune cell populations. Blue and red circles respectively indicate positive and negative association, and the size of each circle corresponds to the correlation coefficient. Red and green lines respectively indicate positive and negative correlation between FAM83A and each immune cell type

The tumor immune microenvironment is a key determinant of patient prognosis. Therefore, we next evaluated the relationship between FAM83A expression levels and the infiltration of 22 immune cell types in LUAD. As shown in Fig. 3, FAM83A expression correlated positively with the infiltration of neutrophils, resting mast cells, activated myeloid dendritic cells (DCs), M2 macrophages, M1 macrophages, M0 macrophages, activated NK cells, resting NK cells, regulatory T cells (Tregs), follicular helper T cells, CD4^+^ memory activated T cells, CD4^+^ naïve T cells, and CD8^+^ T cells (Fig. 3). Conversely, FAM83A exhibited negative correlation with eosinophils, activated mast cells, resting myeloid DCs, monocytes, gamma delta T cells, CD4^+^ resting memory T cells, plasma B cells, memory B cells, and naïve B cells (Fig. 3). In summary, FAM83A may exert an immunomodulatory effect in the LUAD microenvironment, suggesting its potential as a target for lung cancer immunotherapy.

### FAM83A can distinguish between malignant and benign pleural effusion

**Fig. 4:**
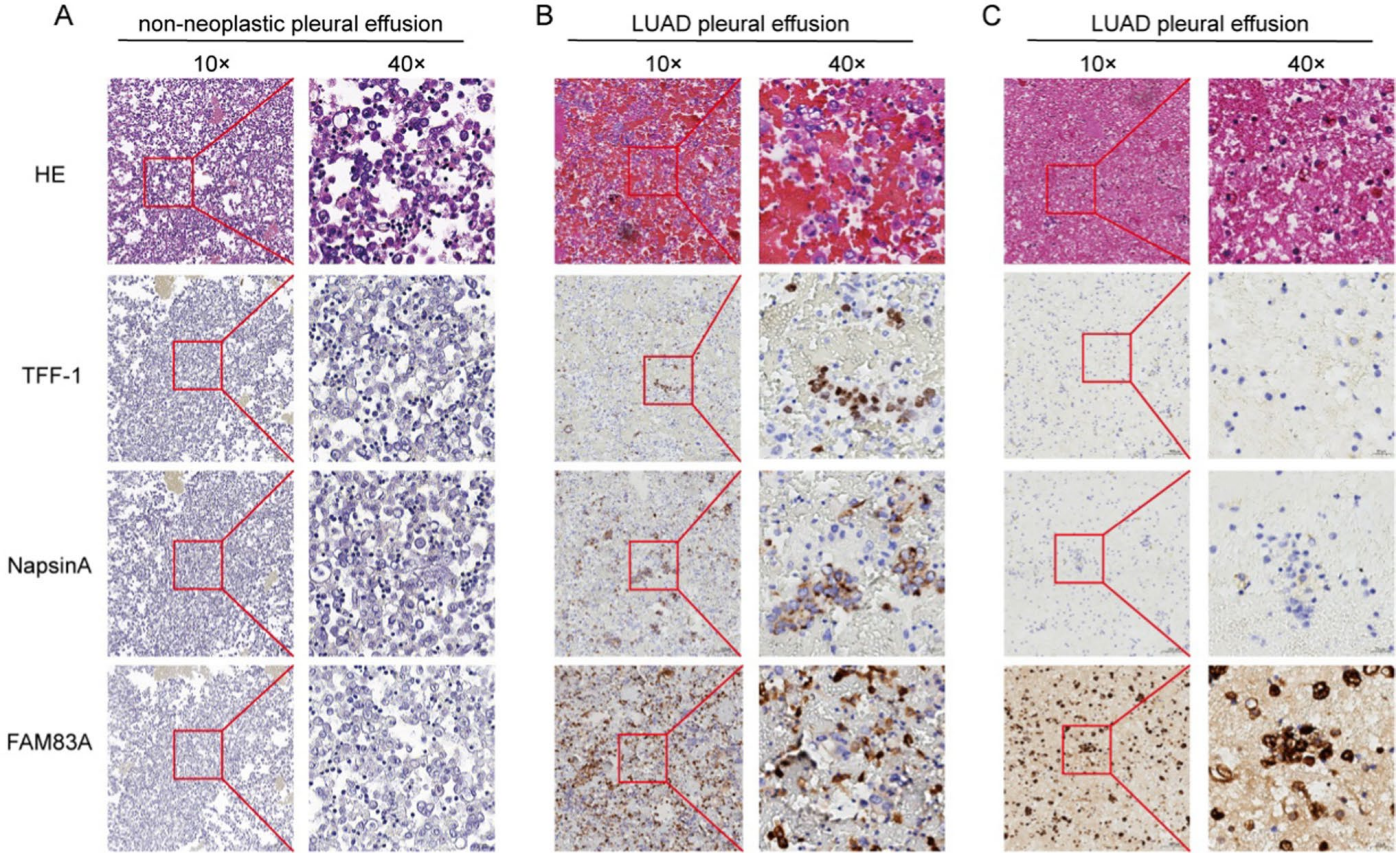
Representative images of immunohistochemical staining of FAM83A, TTF-1 or Napsin A in (**A**) non-neoplastic and (**B, C**) LUAD pleural effusion cell blocks

Based on the findings so far, we hypothesized that FAM83A is a potential diagnostic biomarker for pleural effusion in LUAD patients. To verify our hypothesis, we analyzed the expression of FAM83A, TFF-1 and NapsinA in pleural effusion cell blocks from 40 LUAD patients to 21 non-neoplastic patients through IHC. Positive FAM83A expression was detected in 92.5% of the specimens from LUAD patients, while 95.2% of the non-neoplastic specimens were negative for FAM83A (Table 3). Similar trends were observed for the expression of TTF-1 and NapsinA (Fig. 4A, B). Interestingly, FAM83A staining was more pronounced in some LUAD pleural effusion samples that were weakly positive for TTF-1 or NapsinA (Fig. 4B, C). Taken together, FAM83A is a promising diagnostic biomarker for pleural effusion in LUAD patients.

**Table 3:** FAM83A protein expression in 21 non-neoplastic pleural effusion and 40 LUAD pleural effusion samples

Group	n	Positive	Negative	Positivity rate	χ^2^	*P*
Non-neoplastic pleural effusion	21	1	20	4.8%	45.131	< 0.001
LUAD pleural effusion	40	37	3	92.5%

## Discussion

Malignant pleural effusion (MPE) is frequently observed in patients with lung adenocarcinoma (LUAD), and indicates advanced or progressive stage of the disease. Pleural fluid cytology is a less invasive tool for the diagnosis of MPE, and warrants the identification of effective biomarkers. In the present study, we identified FAM83A as a potential immune-related biomarker of pleural effusion for diagnosing LUAD.

FAM83 family has eight members (FAM83A to FAM83H) that share a highly conserved N-terminal DUF1669 domain [16]. The DUF1669 domain facilitates the interaction between FAM83 members and CK1 isoforms, which in turn regulates cell division and apoptosis [17]. Furthermore, the FAM83 members are overexpressed in many tumors and associated with cancer growth, metastasis, and therapy resistance [16, 18]. FAM83A, the smallest member of this family, has been extensively studied in various cancers. It is upregulated in breast cancer, and portends poor prognosis [19–21] as its contributes to EGFR-TKI resistance by phosphorylating the downstream c-RAF and PI3K p85 [22]. Additionally, FAM83A is overexpressed in HER2-positive breast cancer cells and its knockdown severely inhibits proliferation and induces apoptosis [23]. FAM83A expression is also correlated with the chemoresistance and stemness of triple-negative breast cancer cells [24]. In addition, FAM83A is upregulated in ovarian cancer and promotes tumor progression by activating the Akt/Wnt/β-catenin pathway [25]. Likewise, FAM83A is overexpressed in pancreatic cancer and correlates with worse overall survival and disease-free survival [26] on account of its ability to promote cancer stem cell-like traits and chemoresistance via the WNT/β-catenin and TGF-β signaling pathways [27, 28]. In pancreatic ductal adenocarcinomas, elevated FAM83A expression drives cell survival and tumorigenicity through a MEK/ERK-FAM83A feed-forward loop [29]. The role of FAM83A is ambiguous in cervical cancer, with one study showing a tumor‑suppressive role that involves regulation of integrins [30], whereas others reporting an oncogenic function mediated via PI3K/AKT/mTOR pathway, epithelial mesenchymal transition (EMT) and Wnt signaling pathway [31, 32]. FAM83 induces EMT of head and neck squamous cell carcinoma cells by activating the Wnt/β-catenin signaling pathway, which promotes proliferation and metastasis [33]. Furthermore, FAM83A is upregulated in hepatocellular carcinoma and correlates with poor progression-free survival, and induces migration, invasion and metastasis of the tumor cells by activating EMT signaling and forming a FAM83A/PI3K/AKT/c-JUN positive-feedback loop [34]. Thus, abnormal expression of FAM38A is involved in the development of various cancers.

Liu et al. showed that FAM83A is overexpressed in lung cancer tissues and closely associated with poor survival, and its knockdown significantly suppressed the proliferation, migration and invasiveness of lung cancer cell lines through inactivation of EGFR/MAPK/CHKA signaling [35]. Zheng et al. showed that high expression of FAM83A correlates with advanced TNM stage and poor prognosis in lung cancer, and promotes tumor cell proliferation and invasion by regulating Wnt and Hippo signaling pathways and EMT [36]. Hu et al. found that elevated FAM83A expression in NSCLC is associated with poor prognosis, and FAM83A promotes tumorigenicity at least partly via the ERK and PI3K/Akt/mTOR pathways [37]. Zhou et al. also reported that FAM83A is upregulated in NSCLC, and correlates with metastasis and poor survival. It promotes the migration and invasion of NSCLC cells by inducing EMT via the PI3K/ATK/Snail signaling pathway [38]. Richtmann et al. showed that FAM83A is elevated in NSCLC and associated with poor prognosis, and is involved in proliferation, anchorage-independent growth, migration, and EGFR pathway activation in the tumor cells [39]. Five different cohort studies have shown that LUAD tissues express higher levels of FAM83A compared to adjacent lung tissues, and high FAM83A expression portends poor clinical outcomes such as advanced stage and shorter OS [40–44]. In addition, FAM83A drives PD-L1 expression via ERK signaling, and the co-expression of FAM83A and PD-L1 correlates with poor prognosis in LUAD [43]. Based on these reports, we concluded that FAM83A is highly expressed in lung cancer tissues, and is associated with poor prognosis. Moreover, FAM83A promotes lung oncogenesis through the EGFR/MAPK/CHKA, PI3K/ATK/Snail, Wnt, Hippo, EMT, PI3K/Akt/mTOR, ERK, and EGFR pathways. Thus, FAM83A is a promising biomarker and therapeutic target of lung cancer that warrants further investigation.

FAM83A has been implicated in the modulation of the immune response in lung adenocarcinoma through several intricate mechanisms. Primarily, FAM83A appears to influence immune cell recruitment and activity within the tumor microenvironment by altering cytokine and chemokine profiles. Studies have demonstrated that overexpression of FAM83A is associated with upregulation of pro-tumorigenic cytokines such as IL-6 and TGF-β, which are known to foster an immunosuppressive milieu conducive to tumor growth and progression [43]. Additionally, the interaction of FAM83A with the epithelial-to-mesenchymal transition (EMT) process facilitates the remodeling of the extracellular matrix, creating a physical barrier that impedes immune cell infiltration and enhances the recruitment of immunosuppressive cells such as regulatory T cells (Tregs) and myeloid-derived suppressor cells (MDSCs) [45]. These immunosuppressive cells further exacerbate the immunoevasive properties of the tumor by secreting additional suppressive cytokines and directly inhibiting the function of effector immune cells. Furthermore, bioinformatics analyses of lung adenocarcinoma samples have revealed that high FAM83A expression correlates with reduced infiltration of CD8^+^ T cells and increased infiltration of Tregs, supporting the notion that FAM83A promotes an immunosuppressive tumor microenvironment [40]. Our research shows that FAM83A expression correlated positively with the infiltration of M2 macrophages, M1 macrophages and CD8^+^ T cells. Conversely, FAM83A exhibited negative correlation with resting myeloid DCs, CD4^+^ resting memory T cells, memory B cells, and naïve B cells. Collectively, the multifaceted role of FAM83A in orchestrating an immune landscape that favors tumor evasion and progression in lung adenocarcinoma.

The studies conducted on the role of FAM83A in cancer pathogenesis have largely concentrated on tumor tissue samples. Pleural effusion is a common symptom of advanced LUAD, and the distinction between MPE and benign pleural effusion is essential for determining the optimal treatment regimen. We found that FAM83A is a favorable diagnostic biomarker for pleural fluid cytology in LUAD, and its highly sensitive to MPE as opposed to non-neoplastic pleural effusion. Moreover, FAM83A expression was also higher in LUAD tissues compared to paired normal tissues, which is consistent with other studies and supports FAM83A as a credible biomarker of LUAD biopsies. Although the new IASLC/ATS/ERS LUAD classification has recommended TTF-1 or Napsin A as an immunohistochemical marker for biopsy or cytology specimens [46], the variable sensitivity of these markers may lead to an inappropriate treatment [14]. Therefore, combining FAM83A with TTF-1 or Napsin A may improve diagnostic sensitivity.

In conclusion, FAM83A is a potential immune-associated biomarker in LUAD biopsies and pleural effusion specimens, which provides a new and effective option for pleural effusion cytology and may become a key target in future therapeutic strategies. In-depth study of the specific mechanism of FAM83A in LUAD and its interactions with other molecules could provide a theoretical basis and experimental rationale for the development of new therapeutic approaches.

## Data Availability

The data that support the findings of this study are available in the cBioPortal for Cancer Genomics at http://www.cbioportal.org. The data contains 4 datasets of LUAD-1 (OncoSG, Nat Genet 2020), LUAD-2 (TCGA, Firehose Legacy), LUAD-3 (TCGA, Nature 2014) and LUAD-4 (TCGA, PanCancer Atlas).
